# AIMS: An Automatic Semantic Machine Learning Microservice Framework to Support Biomedical and Bioengineering Research

**DOI:** 10.3390/bioengineering10101134

**Published:** 2023-09-27

**Authors:** Hong Qing Yu, Sam O’Neill, Ali Kermanizadeh

**Affiliations:** School of Computing and Human Sciences Research Centre, University of Derby, Derby DE22 3AW, UK; s.oneill@derby.ac.uk (S.O.); a.kermanizadeh@derby.ac.uk (A.K.)

**Keywords:** AI automation, biomedical, machine learning, microservices, knowledge graph, semantic web services (SWS)

## Abstract

The fusion of machine learning and biomedical research offers novel ways to understand, diagnose, and treat various health conditions. However, the complexities of biomedical data, coupled with the intricate process of developing and deploying machine learning solutions, often pose significant challenges to researchers in these fields. Our pivotal achievement in this research is the introduction of the Automatic Semantic Machine Learning Microservice (AIMS) framework. AIMS addresses these challenges by automating various stages of the machine learning pipeline, with a particular emphasis on the ontology of machine learning services tailored to the biomedical domain. This ontology encompasses everything from task representation, service modeling, and knowledge acquisition to knowledge reasoning and the establishment of a self-supervised learning policy. Our framework has been crafted to prioritize model interpretability, integrate domain knowledge effortlessly, and handle biomedical data with efficiency. Additionally, AIMS boasts a distinctive feature: it leverages self-supervised knowledge learning through reinforcement learning techniques, paired with an ontology-based policy recording schema. This enables it to autonomously generate, fine-tune, and continually adapt to machine learning models, especially when faced with new tasks and data. Our work has two standout contributions demonstrating that machine learning processes in the biomedical domain can be automated, while integrating a rich domain knowledge base and providing a way for machines to have self-learning ability, ensuring they handle new tasks effectively. To showcase AIMS in action, we have highlighted its prowess in three case studies of biomedical tasks. These examples emphasize how our framework can simplify research routines, uplift the caliber of scientific exploration, and set the stage for notable advances.

## 1. Introduction

The fusion of machine learning and biomedical and bioengineering research has brought a paradigm shift in the way we understand, diagnose, and treat an array of health conditions. With rapid advancements in technology and an influx of high-dimensional data, the role of machine learning (ML), and especially AutoML, has become central to the process of knowledge discovery in this field [1]. Providing a machine learning solution often creates a burden for biomedical and bioengineering researchers, who must seek additional support to develop or test different tools for each step of their study. The burgeoning complexity of biomedical research underscores the need for the automatic generation and optimization of machine learning models that can keep pace with this data-driven evolution [2]. The impetus for our research stemmed from challenges faced by our university’s biomedical research group. They grappled with integrating a myriad of tools, algorithms, and domain-specific knowledge in their investigative pursuits. We responded by devising a framework tailored for these exact scenarios. This methodology represents an innovative approach, unprecedented in its application across any other research domain. Such distinctiveness makes our contribution particularly fitting for this special issue on machine learning technology in biomedical engineering. In this paper, we investigate the novel approach of applying an advanced ontology and context enforcement learning approach that can support the automation of a machine learning process for biomedical and bioengineering research. This work has great potential to be applied to other domain areas, but the background ontology requires updating according to the domain knowledge.

The AutoML frameworks provide a robust solution to this rising demand. They offer end-to-end pipelines that encompass all necessary steps from data preprocessing to hyperparameter tuning and model evaluation, automating labor-intensive and error-prone manual tasks [3]. By significantly reducing the time taken for the model development process, they allow researchers to focus on interpreting and applying results, thus accelerating the pace of discovery in the biomedical and bioengineering field.

Despite the undeniable potential of AutoML, several gaps and challenges persist in its implementation in biomedical and bioengineering research. First, biomedical data, with their unique characteristics, including high dimensionality, heterogeneity, and inherent noise, require specialized preprocessing and analytical approaches. Current general-purpose AutoML frameworks may not adequately address these needs. Second, these frameworks often lack interpretability, a crucial requirement in the medical field, where understanding the decision-making process of a model is as essential as its predictive accuracy [4]. Finally, integrating domain knowledge into the AutoML process remains an open challenge, although it could greatly improve the quality of models generated and the applicability of their predictions [5]. Addressing these challenges requires a novel framework that enables machines to learn domain-specific knowledge and apply this knowledge to automate decisions in the AutoML process.

This paper introduces an Automatic Semantic Machine Learning Microservice framework designed to bridge these gaps. We refer to each microservice in our framework as AIMS, and these should be implemented based on domain-specific knowledge. It is tailored to the specific needs of biomedical and bioengineering research and places emphasis on enhancing model interpretability, incorporating domain knowledge, and handling the intricacies of biomedical data. Our proposed framework aims to streamline the research process, augment the quality of scientific exploration, and provide a foundation for significant self-learning AutoML in biomedical research. Additionally, three case studies are tested and discussed at the end.

## 2. Related Work, Limitations, and Technology Background

In the work of the biomedical and bioengineering research community, the most important challenge is to search for different tools that work on different datasets and tasks. Our research also begins by examining existing automation technologies and tools.

### 2.1. Related Work and Current Limitations

There is a multitude of AI tools currently available to aid biomedical research, and various automation frameworks have emerged to streamline the process. For instance, machine learning platforms like Google’s TensorFlow [6] and scikit-learn [7] have been widely utilized in biomedical research for tasks such as image analysis, genomics, and drug discovery.

Google’s AutoML [8], TPOT (a tree-based pipeline optimization tool) [9], and H2O’s AutoML [10,11] are some of the popular AutoML tools used for automating the machine learning pipeline. These platforms optimize the process by automating tasks like data preprocessing, feature selection, model selection, and hyperparameter tuning, which are traditionally labor-intensive and error-prone. AutoML offers expedited results, bypassing much of the manual work involved in traditional machine learning, which is especially advantageous for prototype testing or gauging initial user reactions to AI applications. Moreover, AutoML solutions are less prone to becoming obsolete, as they can stay updated with rapid advancements in AI technology, largely due to the investment capacity of major tech vendors. Additionally, AutoML platforms, being hosted solutions, reduce the overhead of building surrounding infrastructure. TPOT aims to simplify the construction of ML pipelines by merging a versatile expression tree depiction of these pipelines with random search techniques like genetic programming. It leverages the scikit-learn library in Python as its foundation for machine learning functionalities. H2O’s AutoML streamlines the machine learning process by autonomously training and fine-tuning various models within a time frame set by the user. Additionally, H2O incorporates several model interpretability techniques applicable to both AutoML collections and distinct models, such as the leader model. These explanations can be effortlessly produced with a singular function, offering an intuitive means to probe and elucidate the AutoML models.

Other than these general-purpose tools, there are also specialized AI platforms tailored for biomedical research. DeepChem [12], for instance, is a machine learning library specifically designed for drug discovery and toxicology, offering specialized features not available in general-purpose libraries.

However, while these tools have made significant strides in advancing biomedical research, there are several limitations associated with their use.

General-purpose ML and AutoML tools, such as TensorFlow and Google’s AutoML, are not specifically designed for handling the unique characteristics of biomedical data, such as high dimensionality, heterogeneity, and inherent noise. This often necessitates significant manual preprocessing before data can be fed into these tools [13].

Furthermore, these tools often lack interpretability, an essential requirement in biomedical research, where understanding the decision-making process of a model is as important as its predictive accuracy [4].

While specialized tools like DeepChem offer features tailored for biomedical applications, they do not cover the entire spectrum of biomedical research and are limited in their scope. Additionally, the automatic integration of domain knowledge into the machine learning process is an ongoing challenge and is not well addressed by current tools [4].

Therefore, there are many recent discussions on multiple biomedical task handling with self-learning, self-optimizations, and self-configuration processes, such as [14], focusing on data science processing automation with optimization, and [3], focusing on feature selection and model training.

### 2.2. Multiple-Task AI System Research

Making the system automatic by learning the solution knowledge about the different tasks is also challenging. Industrial AI leading research groups such as Google AI and Meta AI understood that data-driven AI technologies have issues with performing complex tasks, for example, creating human conversations with contextual understanding or detecting early signs of disease from images. In addition, data-driven AI is resource-intensive and suffers from algorithm bias [15]. Thus, the multiple-task-enabled AI systems with a knowledge-driven approach present a pathway toward a solution to these problems. Why is it thought that a knowledge-driven approach is necessary and crucial for a multiple-task system? There are two reasons:

- The information acquired from different tasks may present value that can be used as the basis to build new ML models for new tasks without requiring high-cost processing to recapture the same feature characteristics.
- Updating knowledge through validation is a relatively consistent process that will be less prone to bias from noisy data.

Upon completion of this research, two new ideas from Google and Meta were published.

Google presents an experimental process based on knowledge mutation [16,17]. Here, knowledge refers to base neural network transformers. To begin with, the experimental environment contains transformers which can work on different tasks (different image datasets for the classification problems). Then, when a new task arrives, the most related transformer will be triggered to perform a mutation process. The mutation process can edit the base model by inserting a new layer, removing a layer, or doing both according to the performance optimization. In the end, a new mutated adapter is created to enable dealing with a similar task next time. Whenever a new task with a new dataset arrives, the mutation process is executed based on the latest mutated model.

The Meta research group presents a world model approach to acquiring knowledge, very much in the spirit of actor–critic reinforcement learning [18]. The system architecture is a combination of smaller modules—configurator, perception, world model, cost, short-term memory, and actor—that feed into each other. The world model module is responsible for maintaining a model of the world that can then be used to both estimate missing information about the world and predict plausible future states of the world. The perception module will receive signals to estimate the current state of the world and, for a given task, the configurator module will have trained the perception module to extrapolate the relevant signal information. Then, in combination, the perception, world model, cost, short-term memory, and actor modules feed into the configurator module, which configures the other modules to fulfill the goals of the task. Finally, the actor module is handed the optimal action to perform as an action. This has an effect on the real world which the perception module can then capture, which in turn triggers the process to repeat. That is, each action will produce a piece of state-changing knowledge feedback to the world model for continuous learning.

Both Google and Meta’s visions derive from the previous hyperparameter-optimization-based AutoML processes [19]. For example, AutoKeras [20], a neural architecture autosearch framework, is proposed to perform network morphism guided by Bayesian optimization and utilizing a tree-structured acquisition function optimization algorithm. The searching framework selects the most promising Keras implemented NN for a given dataset.

The above experimental results show improvements in tackling complex AI tasks and possible pathways toward human-level AI systems. However, there are two main limitations:

- The knowledge definition is too narrow and only uses the generated neural network as the knowledge limits the capability of recording all valuable outcomes through the learning experience.
- There is no unified knowledge representation structure for knowledge inference (machine thinking).

Do we already have a knowledge representation framework from our AI research over the past 70 years? The answer is yes.

### 2.3. Knowledge Representation and Reasoning

Knowledge representation and reasoning (KRR) are always the core research areas in AI systems [21]. Knowledge representation and reasoning (KRR) is a core area of artificial intelligence (AI) that deals with how to symbolically represent information in a way that a computer system can use to reason about the world. This involves understanding and emulating human-like thinking and the ability to make deductions, inferences, or predictions. KRR aims to enable machines to represent knowledge in a manner that they can reason with, as humans do. Here are the main components:

Knowledge representation (KR): This is about how to store, retrieve, and modify knowledge in an intelligent system. Various paradigms like semantic networks, frames, rules, and ontologies have been developed for this purpose.

Semantic networks: Graph-based structures used to represent knowledge, where nodes represent concepts and edges represent relationships between concepts.

Frames: Data structures for representing stereotypical situations. They contain attributes (or slots) and associated values.

Rules: Represent knowledge in terms of if–then statements.

Ontology: Define a set of representational primitives with which to model a domain of knowledge. Ontologies are used in modern AI applications, especially in the semantic web.

Knowledge reasoning: This is about using the stored knowledge to draw conclusions, make decisions, or infer new knowledge.

The knowledge-enhanced machine learning approach attracts less attention than the data-driven approaches. However, KRR is still key in developing the future generation of AI system development [22]; even Deep Neural Networks (DNNs) can create KRR, just in a different form [23]. In our vision, KRR should not only extract knowledge from data but also learn knowledge from system actions that can support the reasoning process. Knowledge reasoning can be seen as the fundamental building block that allows machines to simulate humankind’s thinking and decision making [24]. With generations of development on KRR, the current most promising approach is the knowledge graph (KG) [24], derived from the semantic web [25] community. A knowledge graph has two layers of representation structure: 1. predefined ontology and vocabularies and 2. instances of triple statements (e.g., dog is animal, where the dog is an instance and is is a predict, while animal is a concept vocabulary defined in the ontology). The reasoning part is to apply the logical side of the ontology, such as description logic (e.g., Is the dog an animal? The reasoning result is ’yes’) [26]. There have been many complex types of ontologies developed in the last decade to solve different KRR problems and applications. The most important development of ontology-driven reasoning is to encode dynamic uncertainty [27], probability [28], and causality [29]. Therefore, the KG-based KRR framework can be applied to implement our proposed vision.

### 2.4. Services and Machine Learning Ontologies

The web services community has researched autoconfiguration or service composition for many years by applying a variety of dynamic integration methods. There are two trends in service composition research:

- Directly extracting the services description file (e.g., WSDL) and Quality of Services (QoS) into a mathematical model with a logical framework for composing services such as a linear logic approach [30] and genetic algorithms [31,32]. The major limitation is that there are no formal specifications for modeling and reasoning. Therefore, the processes are mostly hard-coded to match the logic framework.
- The other trend is to apply semantic web standards for semantically encoding service descriptions and their QoS properties (semantic web services (SWS)) [33,34]. The main benefit is that semantic annotation has an embedded logical reasoning framework to deal with composition tasks.

On the one hand, the semantic web services (SWSs) trend has greater strength for integrating the KRR approach with the same semantic infrastructure and reasoning logic. Currently, there are three standards of OWL-S: composition-oriented ontology, WSMO (task–goal matching-oriented ontology), and WSDL-S (invocation-oriented ontology). On the other hand, there are two differences between our vision’s microservice and normal SWSs. The first one is that AIMS has simpler input and output requirements to perform an efficient composition process. The other is that the purpose of each microservice is to deal with data analytic or machine learning tasks. Therefore, the AIMS ontology needs to be defined by modifying current machine learning ontology standards. Researchers have realized that there is a need to have a machine learning ontology, and some recent proposals in this domain are the Machine Learning Schema and Ontologies (MLSO), which introduces twenty-two top-layer concepts and four categories of lower-layer vocabularies (the detailed ontology design is in [35]), and the Machine Learning Ontology (MLO), which proposes to describe machine learning algorithms with seven top-layer concepts of Algorithm, Application, Dependencies, Dictionary, Frameworks, Involved, and MLTypes [36].

The existing ontology and schema provide a foundational base that can be integrated and augmented to define a more comprehensive schema for generating automotive AI solutions in the biomedical domain. The primary enhancement required is to effectively present the knowledge acquired from each task. Additionally, a self-learning policy is essential to aid machines in comprehending the task context and devising the most optimal pathway to offer a solution.

### 2.5. Generative AI

Recently, generative AI technology, such as ChatGPT and its associated APIs, has marked a significant advancement in AI research. These technologies are primarily designed to engage in text-based conversations, providing solutions to queries and problems in a natural, human-like manner. This form of AI has shown tremendous utility in diverse fields, from customer service to education, demonstrating its versatility.

However, when applied to more complex domains like biomedical research, there are notable limitations. Specifically, the ability of these models to generate code or automated solutions for multistep biomedical problems is limited. The key issue lies in the representation and understanding of data tokens within these problem spaces. In biomedical research, data tokens can represent complex and highly specific biological or medical entities, procedures, or relationships, which can be challenging for AI models to comprehend.

Generative AI models like ChatGPT operate best when dealing with structured data and clear-cut problem domains. Yet, biomedical research often involves dealing with unstructured or semistructured data, highly domain-specific language and concepts, and complex multistep processes.

Another significant challenge for generative AI, particularly in highly specialized fields such as biomedical research, is the integration and expansion of domain-specific human knowledge within the existing large language model.

Generative AI models are usually trained on extensive and diverse datasets, covering a broad range of topics and languages. As a result, they can effectively generate text that mimics human language in many situations. However, these models typically lack the ability to learn continuously or integrate new knowledge once they have been trained. Their knowledge is essentially frozen at the point of their last training update.

This limitation becomes particularly problematic when attempting to apply these models in rapidly advancing fields such as biomedical research, where new discoveries and innovations continually push the boundaries of existing knowledge. As the model cannot natively integrate this new information, it struggles to provide up-to-date and accurate solutions to complex, domain-specific problems. This limitation also extends to learning from user interactions over time, a process which could theoretically allow the model to fine-tune its responses and become more accurate.

Furthermore, the vast and generalized knowledge base of these models can be a double-edged sword. While it allows them to engage with a wide variety of topics, it can also lead to dilution of specialist, domain-specific knowledge. The models may struggle to produce in-depth, nuanced responses to specialized queries due to the sheer breadth of their training data.

In summary, while generative AI has shown significant promise, its limitations in integrating and extending domain-specific human knowledge, coupled with its inability to learn continuously, present considerable challenges for its application in specialized fields like biomedical research. Overcoming these challenges will require novel approaches to model training and updating, making it an exciting area for future AI research and development. A potential strategy could involve using pretrained, domain-specific transformers as a base model. This would facilitate the use of customized small research datasets to efficiently produce a high-quality model. However, this approach necessitates a base model framework to select the most suitable transformer effectively.

### 2.6. The Gaps

By reviewing the current state of the art, we found that there are research gaps remaining to achieve our goal:

- Self-supervised knowledge generation during the machine learning process and solution creation: In the past, knowledge generation system mainly referred to expert systems that acquire knowledge from human expertise or systems that transform existing knowledge from one presentation to the other. Enabling the understanding of common knowledge in the biomedical domain is crucial. Reference [29] presents an automatic process of disease causality knowledge generation from HTML-text documents. However, it still does not fully address the problem of how to automatically learn valuable knowledge from the whole task–solution–evaluation machine learning life cycle. Considering human-level intelligence, we always learn either directly from problem-solving or indirectly through other human expertise (e.g., reading a book or watching a video), or a combination of both (e.g., reflecting on the opinions of others).
- Provisioning a knowledge-guided auto-ML solution: In contrast to the first gap, there are no significant research works on using knowledge to assist in providing an AI solution. Again, compared with human-level intelligence, we always try to apply acquired knowledge or knowledge-based reasoning to solve a problem. We can consider that the transformer process [37] is a step forward in this direction. We can treat well-trained AI models as a type of knowledge to apply to different tasks in a similar problem domain. However, there is still no defined framework that can specify what knowledge is required and how to use the knowledge to find a solution to new tasks [16].

## 3. The Framework Architecture

**Figure 1:**
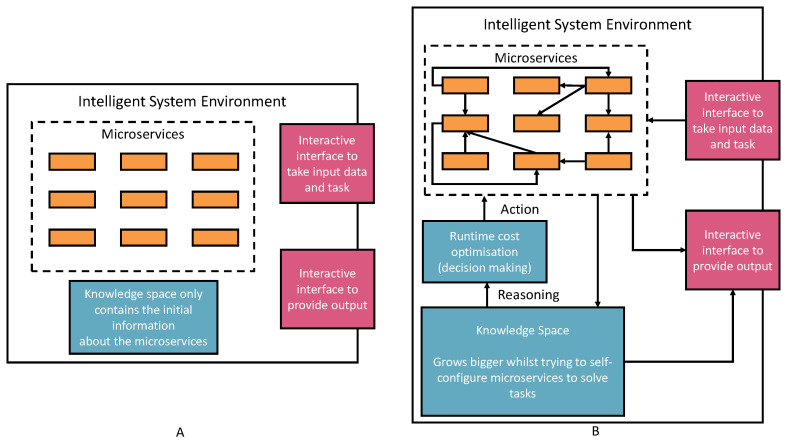
The vision of the self-knowledge learning approach with semantic ML microservices. (**A**) shows the initial environment of the framework without any knowledge learning. (**B**) shows the knowledge growing after learning from the actions that tried to provide an solution to the given tasks.

**Figure 2:**
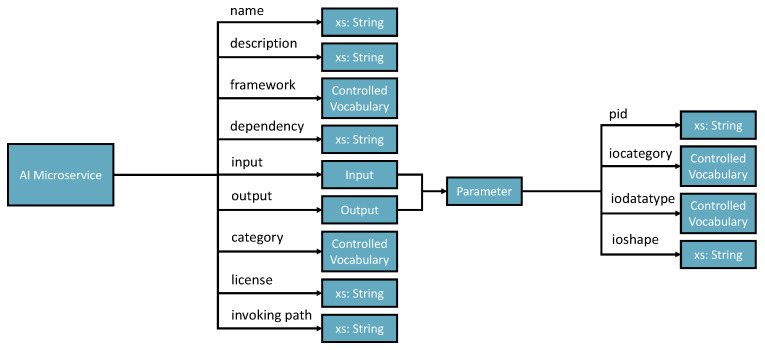
AI Microservice Registration Ontology.

Figure 1 represents our vision of self-supervised knowledge learning with the AIMS engineering approach. The left part of the Figure 1A presents the initial settings of the intelligent environment. The initial environment only contains default AIMS information, such as purposes, I/O requirements, and invokable URI (detailed AIMS metadata ontology is introduced in the next section). However, the initial settings are ready to perform four things:

- Registering new AIMSs (Automatic Semantic Machine Learning Microservices) from outside the environment. The registration process is through the interactive interface according to the defined microservice ontology (see Figure 2). Therefore, human involvement in machine learning microservice engineering is a core part of this vision, which defines humans as educators to teach basic skills and capabilities to deal with different tasks. Then, the environment will reuse these skills and capabilities to acquire knowledge. The knowledge will provide powerful reasoning sources to independently deal with complex tasks, decision making, and creating new pipelines. Specific to the biomedical research, the registration ontology can refer to biomedical engineering ontologies, including Disease Ontology, Foundational Model of Anatomy (FMA), Human Phenotype Ontology (HPO), and many others [38].
- Taking tasks with a variety of inputs, such as CSV data files, images, text, and audio data: The environment autoconfigures on the default AIMSs and provides solutions to the tasks. The success or failure outcome will be recorded as knowledge. Microservice autoconfiguration refers to the automated setup and configuration of individual microservices in a pipeline to serve a machine learning task in our context. The microservice human engineering process will start if there are no suitable AIMSs to deal with the task.
- The environment can compose multiple AIMSs to complete a task if one single microservice cannot achieve it.
- The environment can start learning, representing, and storing knowledge in the knowledge space as knowledge graph data. The knowledge is derived from processing input data, the autoconfiguration process, and task outcomes. The knowledge size will increase and thus provide better optimizations, autoconfiguration, and feedback to the system user.

To realize the vision presented in Figure 1, we discuss the related existing technologies and their research outcomes that can be adapted into our research next.

## 4. Self-Supervised Knowledge Learning for Solution Generation

**Figure 3:**
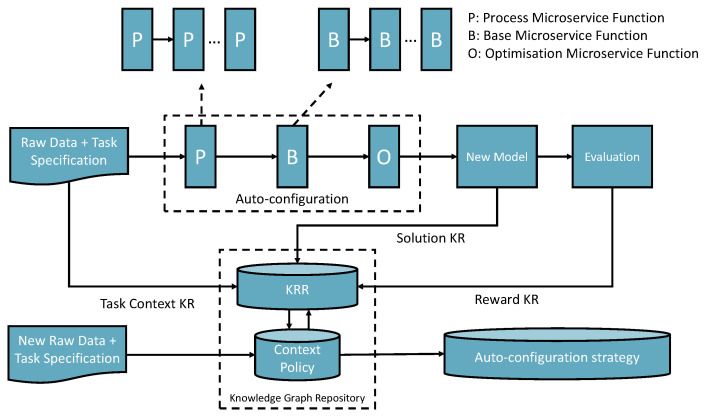
The vision of the self-knowledge learning approach with AI microservices.

The self-supervised knowledge learning approach involves three types of autoconfiguration transfer learning methods. Figure 3 presents the overall learning framework.

The first method is knowledge space searching and transferring: A task with a dataset (referred to as a task-context) arrives, and there is no previous knowledge related to the task-context. Therefore, the knowledge space will be searched to try to find a possible microservice that can match the context to complete the task or search for a pipeline (workflow) that contains multiple I/O compatible AIMSs together towards the best and successful completion which can be optimized. The task context and the optimized solution are recorded as task input and output knowledge. The evaluation will generate rewards for the policy knowledge space (we demo a detailed process in the sections Experimental Implementation and Scenario Evaluation and Lesson Learned). In addition, the knowledge learned from the process will be recorded to update the world knowledge space.

The second one is the mutation of a previously generated context-matched solution (a composition transfer learning process; we demo a detail process in the section Scenario Evaluation and Lesson Learned). If the new task context matches with a previously recorded task context in the knowledge space, then the previous solution will be loaded to adapt to the new datasets and the optimization process. Finally, a new mutated solution is created and recorded as new knowledge with the new evaluation rewards and world knowledge of the KRR environment.

The third one is the continuous learning mutation method based on the reinforcement learning approach. With the growth of the KRR statements, the automutation will take place using world knowledge to retrain the solutions according to the rewards. The third learning method takes place offline only but continues carrying out an update when KRR is updated.

## 5. Experimental Implementation

**Figure 4:**
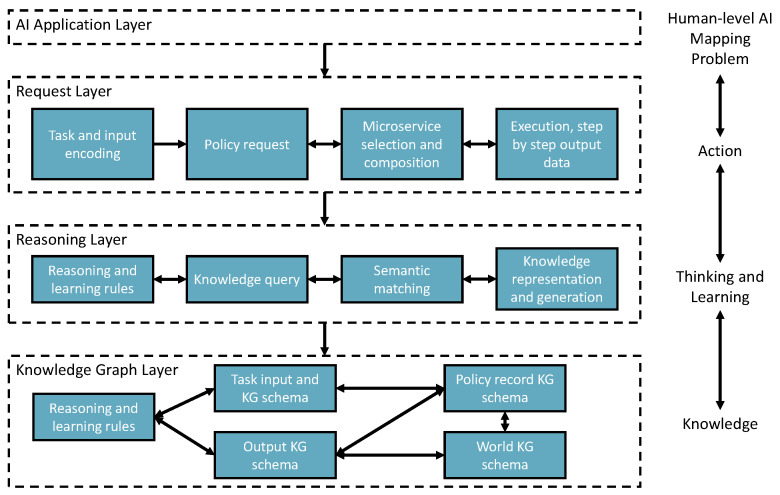
Self-supervised knowledge learning and reasoning framework design.

Figure 4 shows a three-layer implementation of the vision. This structure reflects to our vision that AI system should have three major capabilities of learning knowledge, reasoning (thinking), and reacting to the problem. The figure also shows how these layers map to the automotive solution provisioning process.

- The request layer takes tasks and inputs from AI applications to trigger the solution searching and self-learning processes. Task context is semantically encoded to enable starting the policy knowledge to explore the environment for learning, creating, or finding solutions.
- The reasoning layer takes the request layer’s semantic reasoning tasks for semantic matching, reasoning, and performing the reinforcement learning mechanism. Finally, the policy will be recorded in the knowledge graph layer. In addition, the newly added AIMSs are registered to the environment with semantic annotations through knowledge registration and generation components.
- The knowledge graph layer remembers the knowledge data in the knowledge graph triple store based on different types of knowledge schemata.

### 5.1. Knowledge Ontology Implementation

AIMS registration ontology defines nine parameters (see Figure 2).

- Name— Must be no duplication in the system, and the registration process will check the name’s legibility.
- Description—A short presentation of the AIMS for human understanding.
- Framework—Indicates the programming framework used to develop the AIMS. Normally, it should be just one framework as AIMS is designed to be decoupled and ideally single responsibility.
- Dependency—Describes the required programming libraries that need to be preconfigured to enable the AIMS to work. The schema includes id, library install port URI and version.
- Input and output—Specifies the parameters that should be in the input and output messages.
- Category—Tells what AI-related domain the ms works on, such as supervised classification, unsupervised clustering, image classification base model, and more.
- License—Identifies the use conditions and copyright of the AIMS.
- Invoke path—Contains the portal for accessing the AIMS. The path can be a local path or URI of a restful API.

Each given task triggers a context knowledge creation that collects knowledge of the following:

- The type of input data—A controlled variable that majorly includes normal dataset (e.g., tableau data stored in a CSV file, image, or text).
- Task domain—Free text to record the specific application domain.
- Desire output type—Records the output required to complete the task successfully.
- The parameters—The dataset or data presents the initial characters of the input data. For example, the number of columns and column names of the tableau data will be remembered as part of the context knowledge of the task.

The output of the performed task can be categorized into two types, failure and success. Both failure and success need to update the policy knowledge link to the task-input context. Failure has no solution registered to the knowledge but records which AIMSs have been successfully invoked (can be an empty list) until the step that cannot continue going further. So, the failure experience will tell the system administrators (people) what AIMS(s) are required to create a solution. The success registers the solution location and changes the policy with the reward value. If the solution contains a workflow of composed AIMSs, then the workflow will also be registered as knowledge with the normalized rewards for each of the AIMSs.

**Figure 5:**
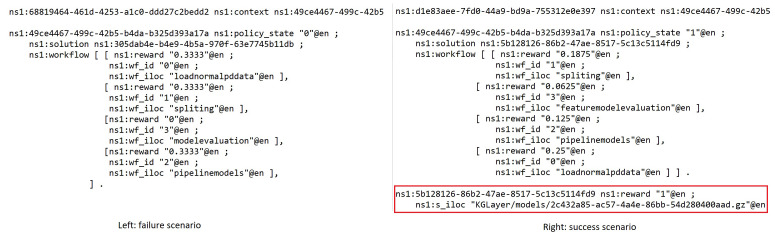
Reinforcement learning policy generation for the knowledge layer.

The policy ontology is designed as follows:

- Policy context—Links to a task-context.
- Policy state—1 is success and 0 is failure, the binary state only presents whether the whole workflow is work or not but inside the workflow context that shows the continuous measure of individual component’s potential contribution toward to success in other possible solutions (see Figure 5).
- Solution iloc—The location where the solution can be loaded and executed.
- Workflow—Presents a pipeline solution that composes multiple AIMSs.
- Solution reward—The reward value stored for the policy that can be the recommended guidance for supporting the creation of a new task solution.

The can be rewarded in a failure pipeline if the individual step is invoked and running successfully. Therefore, the rewarded micorservice can be reused when searching for the alternative success composition solution. Figure 5 shows that failure workflow at run-time provide one of three rewards to the successful individual microservices, but no final solution model is created comparing it with the second searching which created a successful workflow pipeline and recalculated rewards to all four microservices. The successful workflow will reward the total reward value 1 divided by the number of microservices (n) involved (n—order number).

The world knowledge ontology presents the facts learned from the task solution creation process and outputs. There are three types of world knowledge recorded in the current environment:

- Feature optimization outcomes—The features selected in the optimization are valuable, and these features will be reused to create a classification model if the new dataset features are the same.
- Answers for a certain text topic—A generated text answer for a question. The answer quality will be reported as a reward value feedback from humans back to the policy knowledge.
- Image RBG vectors—Map to a classification label. The reward process is the same as the answers.

More world knowledge can be expanded in the environment. By having these commonsense and policy records, reinforcement can be performed to improve the solution accuracy incrementally.

### 5.2. Environment Initialization

The experiment environment is developed by Python in a local single-computer environment. We simplified the AIMS as a .py module in the environment to be invoked and registered. We initialized the environment with three types of AIMSs:

1. Data processing AIMSs that include CSV files to a Panda service, data training and split services, data quality control services, data normalization services, image process services, and data quality control services.
2. ML AIMSs that include clustering services, classification services, GPT-neo-1.3B text generation services [39,40], ViT image classification transformers [41], and Seanborn visualization services.
3. RFECV optimization services.

## 6. Scenario Evaluation and Lessons Learned

### 6.1. Heart Disease Classification Scenario

**Figure 6:**
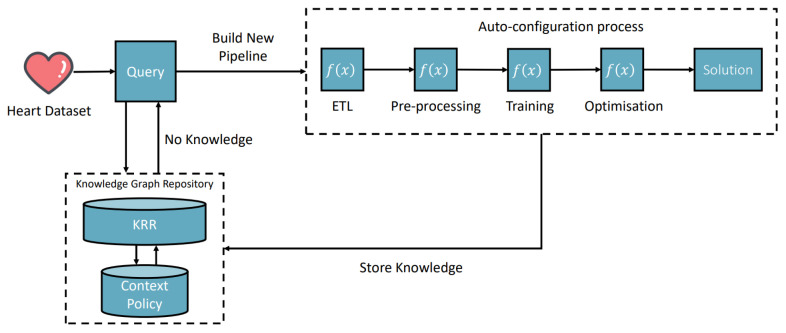
Scenario 1—Heart disease classification solution building and knowledge learning process.

Figure 6 presents one of our use-case scenarios in the medical domain. The task context is as follows:

- Input: 335 clinical CSV heart disease files labeled 0 (no disease) and 1 (confirmed disease).
- Domain: Medical.
- Desire output: An optimized classification pipeline model.

Additionally, the columns of the data are as follows:

- Age: The person’s age in years.
- Sex: The person’s sex (1 = male, 0 = female).
- cp: Chest pain type—value 0: asymptomation, value 1: atypical angina, value 2: nonanginal pain, value 3: typical angina.
- trestbps: The person’s resting blood pressure (mm Hg on admission to the hospital).
- chol: The person’s cholesterol measurement in mg/dL.
- fbs: The person’s fasting blood sugar (>120 mg/dL, 1 = true; 0 = false).
- restecg: Resting electrocardiographic results.
- thalach: The person’s maximum heart rate achieved.
- exang: Exercise-induced angina (1 = yes; 0 = no).
- oldpeak: ST depression induced by exercise relative to rest (ST relates to positions on the ECG plot.
- Slope: The slope of the peak exercise ST segment—0: downsloping; 1: flat; 2: upsloping.
- ca: The number of major vessels (0–3).
- thal: A blood disorder called thalassemia.
- target Heart disease (1 = no, 0 = yes).

The application domain is medical, and the desired output is an optimized classification pipeline model. Figure 6 illustrates the process. The procedure commences with the input of a CSV file containing the Heart Disease dataset, accompanied by a query pertaining to the generation of a predictive model. Following this, the framework attempts to retrieve relevant knowledge via the request layer and its associated functions, as shown in Figure 4. Given the absence of pre-existing knowledge, the system initiates a reasoning process through the reasoning layer. This layer, utilizing the knowledge representation of existing microservices, crafts a workflow. The formulated workflow incorporates four AI microservices: data loading from the CSV, data partitioning, creation of a classification pipeline, and optimization. The culmination of this process results in a model boasting an accuracy rate of 96.8%. Throughout the procedure, various knowledge components are assimilated and documented within the system’s environment. The unique insight derived from the general knowledge context is that eight features are deemed more significant than other columns in determining the classification results. These features are sex, cp, thalach, exang, oldpeak, slope, ca, and thal. The reason these columns are highlighted as the most crucial is because the optimization microservice identified them in generating the most accurate model.

### 6.2. Parkinson Disease Classification Scenario

The second task context is as follows:

- Input: CSV Parkinson’s disease clinical example data with labels 0 (no disease) and 1 (confirmed disease).
- Domain: Medical.
- Desired output: An optimized classification pipeline model.

**Figure 7:**
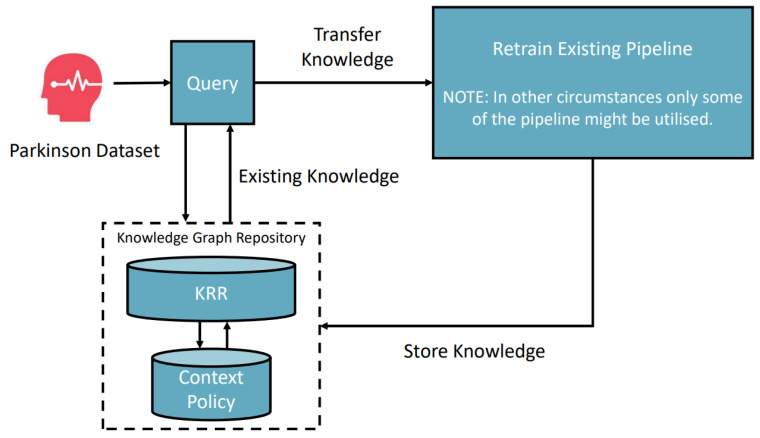
Scenario 2—Parkinson’s disease pipeline transfer classification process.

Figure 7 depicts the scenario in which a similar task of classifying Parkinson’s disease is fed in; the framework starts searching for a solution. As the system environment has preknowledge, gained through the previous heart disease classification, and since the only difference is the dataset when compared with the heart disease classification context, the framework can use the classification pipeline and retrain it to be optimized for the new dataset. We can call this process a composition transfer learning process. The novelty is that the system environment can solve different tasks by applying contextual knowledge of the problem. Thus, the framework can automatically deal with all types of data if the required models are semantically registered in the framework. Through this composition transfer learning, the whole automatic pipeline can produce a 94.6% accurate model.

### 6.3. A Complex Scenario: Mouse Brain Single-Cell RNASeq Downstream Analysis

**Figure 8:**
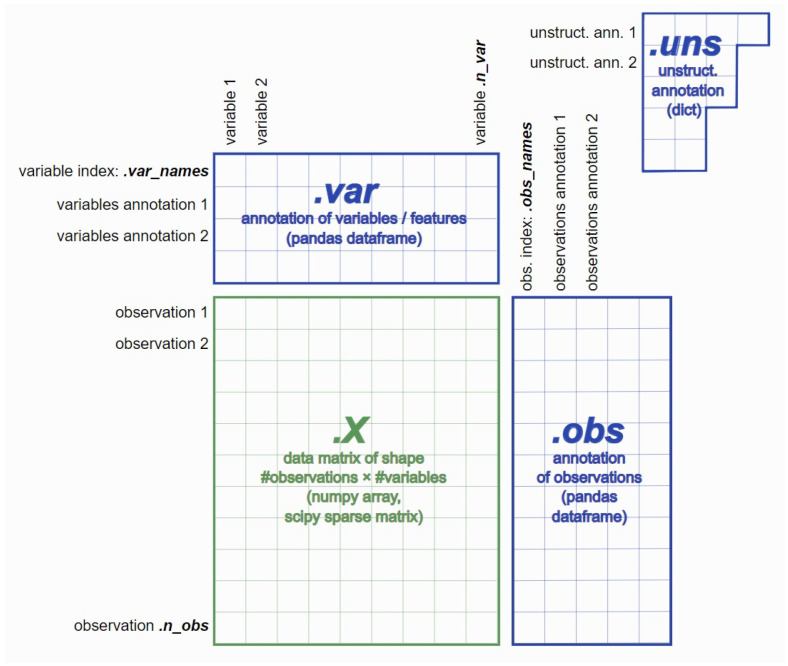
AnnData Structure.

In this section, we use a clustering analysis case study to highlight how the proposed framework can solve a real-world downstream single-cell data analysis task. The clustering analysis of single-cell data offers a powerful tool for a myriad of applications, ranging from understanding basic biological processes to the development of clinical strategies for treating diseases. The clustering analysis task works on a mouse brain single-cell RNASeq dataset. The dataset is publicly available through a workshop tutorial at [42]. There are five sequential processing and analysis steps:

1. Data semantic transforming and loading: For instance, applying AnnData structure [43], where AnnData stores observations (samples) of variables/features in the rows of a matrix (see Figure 8).
2. Data quality control: This aims to find and remove the poor-quality cell observation data which were not detected in the previous processing of the raw data. The low-quality cell data may potentially introduce analysis noise and obscure the biological signals of interest in the downstream analysis.
3. Data normalization: Dimensionality reduction and scaling of the data. Biologically, dimensional reduction is valuable and appropriate since cells respond to their environment by turning on regulatory programs that result in the expression of modules of genes. As a result, gene expression displays structured coexpression, and dimensionality reduction by the algorithm such as principle component analysis can group these covarying genes into principle components, ordered by how much variation they explained.
4. Data feature embedding: Further dimensionality reduction using advanced algorithms, such as t-SNE and UMAP. They are powerful tools for visualizing and understanding big and high-dimensional datasets.
5. Clustering analysis: Groups cells into different clusters based on the embedded features.

Based on the above five steps, we developed four extra microservices, which include AnnData loading, two feature embedding services (t-SNE and UMAP), and clustering services (Louvain graphical clustering algorithms). The other existing microservices which can be involved should be different types of normalization (PCA or CPM algorithm) and K-mean clustering algorithms.

**Figure 9:**
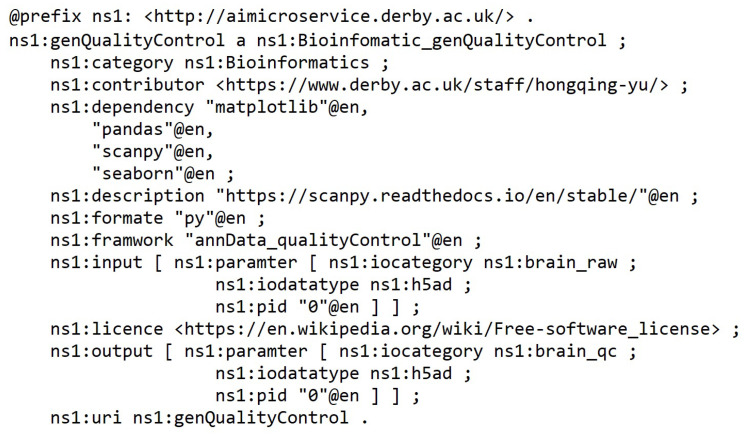
Quality control microservice semantic description.

The microservices were semantically registered into the framework through the interface. Figure 9 depicts an example of a quality control microservice semantic description in the knowledge graph repository.

With all the microservices registered, researchers can start expressing the analysis task to stop, interact, and provide feedback at any stage during the process of automatically creating the solution. The researchers can also see visualizations of outputs produced by different steps. Therefore, researchers can provide preferences for selecting microservices if there are options.

**Figure 10:**
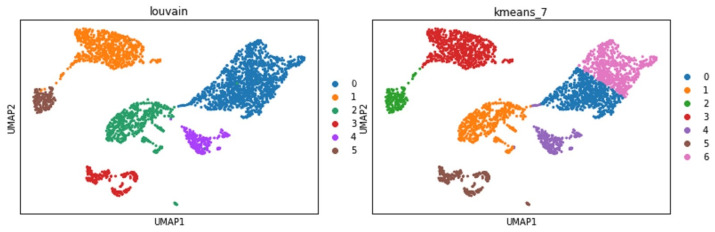
Two clustering outcomes from automatic processes.

A realistic example is that a researcher can specify a clustering task applied to the mouse brain single-cell RNASeq dataset. The framework will first try to see if a single microservice can complete this task. The answer is ’no’, because no semantic-matched microservice can take the RNASeq CSV input and provide the clustering output. At this juncture, the microservice that can take the RNASeq CSV will be invoked to process the data into the next step with the output of AnnData. If there are multiple choices in the composition sequence, all possibilities will be invoked to run, unless the previous knowledge in the policies has a priority. The possibilities have multiple solutions at the end for researchers to analyze in order to give professional feedback to the system. The feedback will help greatly with the knowledge graph policies. For example, suppose the researcher gives feedback to the system that UMAP is the better embedding method than t-SNE but has no priority on the clustering methods. In that case, the framework will produce two possible clustering results, shown in Figure 10.

## 7. Discussion

By evaluating the performance of the test scenarios, we believe the combination of KRR and automation of AIMSs offers a viable strategy for developing human-level AI systems. We created an environment with AIMSs capable of handling text, CSV files, and images as default settings. This environment supports data splitting, classification, prediction, and optimization AIMSs. These findings suggest the system’s capability to generate and optimize solutions for various tasks by applying or creating knowledge. However, there remain some challenges that future work needs to address:

- The advantage of using a triple KG structure to encode KRR elements lies in its unification, standardization, and adaptability across diverse applications. Nonetheless, as the KG expands, its referencing efficiency diminishes, particularly with intricate graph queries. This inefficiency is exacerbated when different knowledge types are stored separately, making union queries on the graph resource-intensive. A proposed solution is to embed the knowledge graph into a more efficient vector space [44]. To achieve this, we plan on investigating state-of-the-art embedding techniques, such as graph neural networks, that can maintain the relationships between entities while offering efficient querying.
- The current system architecture does not support multimodal inputs pertaining to a singular task (multimodal machine learning). While humans can seamlessly integrate visual, auditory, and other sensory data to accomplish tasks, machines struggle to synthesize multiple data types [45,46,47]. Moving forward, we aim to explore fusion techniques, both at the feature and decision levels, to facilitate more comprehensive input processing.
- During the initial stages of our manuscript’s preparation, Google released research papers detailing the mutation of neural networks (NNs) to handle diverse image classification tasks [16,17]. These papers have illuminated the potential of not just mutating data or services but also the possibility of adding or removing NN hidden layers as a form of knowledge storage for future considerations. Our intent is to delve deeper into the dynamics of such mutations and explore frameworks that allow for flexible and dynamic architectural changes in neural networks.

## 8. Conclusions

Our proposed Automatic Semantic Machine Learning Microservice (AIMS) framework presents a novel approach to managing the complex demands of machine learning in biomedical and bioengineering research. The AIMS framework utilizes a self-supervised knowledge learning strategy to ensure automatic and dynamic adaptation of machine learning models, making it possible to keep pace with the evolving nature of biomedical research. By placing emphasis on model interpretability and the integration of domain knowledge, the framework facilitates an improved understanding of the decision-making process, enhancing the relevance and applicability of the generated models. A significant finding of this research is our demonstration that knowledge-based systems can play a pivotal role in self-learning AI systems for biomedical research. Such systems offer the capability to store domain-specific knowledge with reusability and bolster the reinforcement learning processes for machines. Furthermore, the potential of these systems extends beyond biomedical research, suggesting applicability to AI applications in other domains.

The three case studies presented underscore the framework’s effectiveness in various biomedical research scenarios, demonstrating its capacity to handle different types of data and research questions. As such, the AIMS framework not only offers a robust solution to current challenges in biomedical and bioengineering research but also sets a promising direction for future developments in automated, domain-specific machine learning. Further studies are required to evaluate the AIMS framework’s performance across a wider range of biomedical and bioengineering applications and to refine its capabilities for even more efficient and precise knowledge discovery.

## Data Availability

The full implementation and publicly available data used in this project can be found in GitHub repository (https://github.com/semanticmachinelearning/AISMK, accessed on 20 August 2023).

## References

[1] Obermeyer Z., Emanuel E.J. (2016). Predicting the future—Big data, machine learning, and clinical medicine. N. Engl. J. Med..

[2] Miotto R., Wang F., Wang S., Jiang X., Dudley J.T. (2017). Deep learning for healthcare: Review, opportunities and challenges. Briefings Bioinform..

[3] Waring J., Lindvall C., Umeton R. (2020). Automated machine learning: Review of the state-of-the-art and opportunities for healthcare. Artif. Intell. Med..

[4] Holzinger A., Langs G., Denk H., Zatloukal K., Müller H. (2019). Causability and explainability of artificial intelligence in medicine. Wiley Interdiscip. Rev. Data Min. Knowl. Discov..

[5] Zheng W., Lin H., Liu X., Xu B. (2018). A document level neural model integrated domain knowledge for chemical-induced disease relations. BMC Bioinform..

[6] Abadi M., Agarwal A., Barham P., Brevdo E., Chen Z., Citro C., Corrado G.S., Davis A., Dean J., Devin M. (2015). TensorFlow: Large-Scale Machine Learning on Heterogeneous Systems. tensorflow.org.

[7] Pedregosa F., Varoquaux G., Gramfort A., Michel V., Thirion B., Grisel O., Blondel M., Prettenhofer P., Weiss R., Dubourg V. (2011). Scikit-learn: Machine Learning in Python. J. Mach. Learn. Res..

[8] Google LLC (2021). Google Cloud AutoML. https://cloud.google.com/automl/docs.

[9] Le T.T., Fu W., Moore J.H. (2020). Scaling tree-based automated machine learning to biomedical big data with a feature set selector. Bioinformatics.

[10] H2O.ai (2023). H2O AutoML. https://docs.h2o.ai/h2o/latest-stable/h2o-docs/automl.html.

[11] LeDell E., Poirier S. H2O AutoML: Scalable Automatic Machine Learning. Proceedings of the 7th ICML Workshop on Automated Machine Learning (AutoML).

[12] Ramsundar B., Eastman P., Walters P., Pande V. (2019). Deep Learning for the Life Sciences: Applying Deep Learning to Genomics, Microscopy, Drug Discovery, and More.

[13] He J., Baxter S.L., Xu J., Xu J., Zhou X., Zhang K. (2019). The practical implementation of artificial intelligence technologies in medicine. Nat. Med..

[14] Mustafa A., Rahimi Azghadi M. (2021). Automated Machine Learning for Healthcare and Clinical Notes Analysis. Computers.

[15] Ntoutsi E., Fafalios P., Gadiraju U., Iosifidis V., Nejdl W., Vidal M.E., Ruggieri S., Turini F., Papadopoulos S., Krasanakis E. (2020). Bias in data-driven artificial intelligence systems—An introductory survey. WIREs Data Min. Knowl. Discov..

[16] Gesmundo A., Dean J. (2022). An Evolutionary Approach to Dynamic Introduction of Tasks in Large-scale Multitask Learning Systems. arXiv.

[17] Gesmundo A., Dean J. (2022). muNet: Evolving Pretrained Deep Neural Networks into Scalable Auto-tuning Multitask Systems. arXiv.

[18] LeCun Y. (2022). A Path Towards Autonomous Machine Intelligence. Open Review. https://openreview.net/pdf?id=BZ5a1r-kVsf.

[19] Yao Q., Wang M., Escalante H.J., Guyon I., Hu Y., Li Y., Tu W., Yang Q., Yu Y. (2018). Taking Human out of Learning Applications: A Survey on Automated Machine Learning. arXiv.

[20] Jin H., Song Q., Hu X. (2018). Auto-Keras: An Efficient Neural Architecture Search System. arXiv.

[21] Sharma L., Garg P.K. (2021). Knowledge representation in artificial intelligence: An overview. Artificial Intelligence.

[22] Cozman F.G., Munhoz H.N. (2021). Some thoughts on knowledge-enhanced machine learning. Int. J. Approx. Reason..

[23] Hu Z., Yang Z., Salakhutdinov R., Xing E. Deep neural networks with massive learned knowledge. Proceedings of the 2016 Conference on Empirical Methods in Natural Language Processing.

[24] Chen X., Jia S., Xiang Y. (2020). A review: Knowledge reasoning over knowledge graph. Expert Syst. Appl..

[25] Berners-Lee T., Hendler J., Lassila O. (2001). The semantic web. Sci. Am..

[26] Baader F., Horrocks I., Lutz C., Sattler U. (2017). Introduction to Description Logic.

[27] Zhang Q. (2015). Dynamic Uncertain Causality Graph for Knowledge Representation and Probabilistic Reasoning: Directed Cyclic Graph and Joint Probability Distribution. IEEE Trans. Neural Netw. Learn. Syst..

[28] Botha L., Meyer T., Peñaloza R. (2021). The Probabilistic Description Logic. Theory Pract. Log. Program..

[29] Yu H.Q., Reiff-Marganiec S. (2022). Learning Disease Causality Knowledge From the Web of Health Data. Int. J. Semant. Web Inf. Syst. (IJSWIS).

[30] Zhao X., Liu E., Yu H.Q., Clapworthy G.J. (2015). A Linear Logic Approach to the Composition of RESTful Web Services. Int. J. Web Eng. Technol..

[31] Allameh Amiri M., Serajzadeh H. QoS aware web service composition based on genetic algorithm. Proceedings of the 2010 5th International Symposium on Telecommunications.

[32] Qiang B., Liu Z., Wang Y., Xie W., Xina S., Zhao Z. (2018). Service composition based on improved genetic algorithm and analytical hierarchy process. Int. J. Robot. Autom..

[33] Yu H.Q., Zhao X., Reiff-Marganiec S., Domingue J. Linked Context: A Linked Data Approach to Personalised Service Provisioning. Proceedings of the 2012 IEEE 19th International Conference on Web Services.

[34] Dong H., Hussain F., Chang E. (2013). Semantic Web Service matchmakers: State of the art and challenges. Concurr. Comput. Pract. Exp..

[35] Publio G.C., Esteves D., Lawrynowicz A., Panov P., Soldatova L.N., Soru T., Vanschoren J., Zafar H. (2018). ML-Schema: Exposing the Semantics of Machine Learning with Schemas and Ontologies. arXiv.

[36] Braga J., Dias J., Regateiro F. (2020). A machine learning ontology. Frenxiv Pap..

[37] Tan C., Sun F., Kong T., Zhang W., Yang C., Liu C., Kůrková V., Manolopoulos Y., Hammer B., Iliadis L., Maglogiannis I. (2018). A Survey on Deep Transfer Learning. Proceedings of the Artificial Neural Networks and Machine Learning–ICANN 2018: 27th International Conference on Artificial Neural Networks, Rhodes, Greece, 4–7 October 2018.

[38] Filice R.W., Kahn C.E.J. (2021). Biomedical Ontologies to Guide AI Development in Radiology. J. Digit. Imaging.

[39] Black S., Leo G., Wang P., Leahy C., Biderman S. (2021). GPT-Neo: Large Scale Autoregressive Language Modeling with Mesh-Tensorflow (1.0). Zenodo.

[40] Gao L., Biderman S., Black S., Golding L., Hoppe T., Foster C., Phang J., He H., Thite A., Nabeshima N. (2020). The Pile: An 800GB Dataset of Diverse Text for Language Modeling. arXiv.

[41] Bhojanapalli S., Chakrabarti A., Glasner D., Li D., Unterthiner T., Veit A. Understanding Robustness of Transformers for Image Classification. Proceedings of the IEEE/CVF International Conference on Computer Vision (ICCV).

[42] Luecken M.D., Theis F.J. (2019). Current best practices in single-cell RNA-seq analysis: A tutorial. J. Mol. Syst. Biol..

[43] Cannoodt R. (2022). Anndata: ‘Anndata’ for R. https://anndata.readthedocs.io/en/latest/.

[44] Le T., Huynh N., Le B., Farkaš I., Masulli P., Otte S., Wermter S. (2021). Link Prediction on Knowledge Graph by Rotation Embedding on the Hyperplane in the Complex Vector Space. Proceedings of the Artificial Neural Networks and Machine Learning–ICANN 2021: 30th International Conference on Artificial Neural Networks, Bratislava, Slovakia, 14–17 September 2021.

[45] Ramachandram D., Taylor G.W. (2017). Deep multimodal learning: A survey on recent advances and trends. IEEE Signal Process. Mag..

[46] Baltrušaitis T., Ahuja C., Morency L.P. (2018). Multimodal machine learning: A survey and taxonomy. IEEE Trans. Pattern Anal. Mach. Intell..

[47] Li J., Hong D., Gao L., Yao J., Zheng K., Zhang B., Chanussot J. (2022). Deep learning in multimodal remote sensing data fusion: A comprehensive review. arXiv.

